# 3D-printed magnesium/strontium-co-doped calcium silicate scaffolds promote angiogenesis and bone regeneration through synergistic bioactive ion stimulation

**DOI:** 10.1186/s13036-025-00528-6

**Published:** 2025-06-21

**Authors:** Chia-Che Ho, Tuan-Ti Hsu, Yung-Cheng Chiu, Yen-Hong Lin, Pei-Cheng Xie, Chen-Ying Wang

**Affiliations:** 1 Department of Bioinformatics and Medical Engineering, Asia University, Taichung, 41354 Taiwan; 2 High Performance Materials Institute for x-Dimensional Printing, Asia University, Taichung, 41354 Taiwan; 3 Research & Development Center for x-Dimensional Extracellular Vesicles, China Medical University Hospital, Taichung, 404332 Taiwan; 4 School of Medicine, China Medical University, Taichung, 406040 Taiwan; 5 Department of Orthopedic Surgery, China Medical University Hospital, Taichung, 404332 Taiwan; 6 Department of Biomedical Engineering, China Medical University, Taichung, 406040 Taiwan; 7 Graduate Institute of Clinical Dentistry, School of Dentistry, National Taiwan University, Taipei, 106319 Taiwan; 8 Division of Periodontics, Department of Dentistry, National Taiwan University Hospital, Taipei, 106319 Taiwan

**Keywords:** 3D printing, Calcium silicate, Bone regeneration, Magnesium, Strontium

## Abstract

Bone defects resulting from trauma, infection, or surgical resection require biomaterials that support osteogenesis and vascularization for effective regeneration. In this study, we developed a 3D-printed magnesium- and strontium-co-doped calcium silicate (MSCS) scaffold using direct ink writing to optimize its bioactivity and structural integrity. X-ray diffraction confirmed the successful incorporation of Sr and Mg, leading to phase modifications that influenced ion release and degradation. Wettability and mechanical testing showed that Sr improved the stability, while Mg accelerated degradation, with M5S5 co-doping exhibiting a balanced degradation profile. In vitro, Wharton’s jelly mesenchymal stromal cells cultured on M5S5 scaffolds displayed enhanced proliferation, cytoskeletal organization, and osteogenic differentiation, as evidenced by increased alkaline phosphatase activity and bone matrix protein expression. Angiogenesis assays using human umbilical vein endothelial cells revealed that Sr and Mg co-doping synergistically enhanced vascular endothelial growth factor and angiopoietin-1 secretion, thereby promoting endothelial tube formation. In vivo micro-computed tomography and histological analysis of a rabbit femoral defect model confirmed that M5S5 facilitated extensive new bone formation, exhibiting superior trabecular architecture and mineralization. These findings highlight MSCS scaffolds as promising biomaterials for bone tissue engineering applications.

## Introduction

Bone defects, which are frequently encountered in clinical settings, present a significant challenge because of their diverse etiologies, including trauma, infection, tumor resection, and congenital anomalies [1, 2]. These defects compromise skeletal integrity and function, leading to pain, limited mobility, and potential disabilities [3]. External biomaterials are essential for promoting bone tissue regeneration when defects exceed the self-repair capacity of the body [4]. Traditional bone repair methods, such as autologous bone grafting and allografting, have limitations. Although biocompatible, autografts result in donor site morbidity and limited graft availability [5]. Meanwhile, allografts carry the risks of immune rejection and disease transmission. These limitations have driven the search for safer and more effective bone repair alternatives [6, 7].

Bioceramics are widely used in bone grafting owing to their biocompatibility [8]. Recently, calcium silicate-based ceramics (CS) have garnered significant attention as novel bioactive alternatives for bone tissue engineering [9–11]. Compared to calcium phosphates (CaPs), CS promotes faster formation of bone-like hydroxyapatite in physiological environments and exhibits superior osteoconductivity [12]. Studies have also demonstrated the advantages of promoting mesenchymal stem cell proliferation and differentiation. CS are characterized by their ability to continuously release ions that play crucial roles in regulating bone regeneration. For instance, released Si ions (Si) positively affect cell proliferation and subsequent bone formation. Additionally, calcium ions (Ca) released from CS scaffolds react with PO_4_^3−^ ions in biological fluids, leading to the mineralization of a hydroxyapatite layer on the preformed SiO_2_ surface of the scaffold. This hydroxyapatite layer facilitates subsequent tissue integration with the scaffold.

Recent advances in bone-substitute materials have emphasized the integration of ion efficiency as a key design strategy [13]. Numerous studies have highlighted the biological effects of divalent cations such as Ca^2+^, Sr^2+^, and Mg^2+^, which significantly influence cell adhesion, migration, and differentiation. The incorporation of functional ions into biomaterials for a controlled local release has emerged as a promising and cost-effective approach for enhancing bone regeneration. Unlike the potential instability of loaded cytokines, proteins, or drugs, this strategy maintains chemical stability while offering therapeutic benefits. Mg^2+^ ions are essential for maintaining the physiological homeostasis of tissues and organs and promoting angiogenesis [14]. Studies have shown that specific ranges of high Mg concentration can enhance the angiogenic capability of stem cells. Mg can also induce endothelial cells to produce nitric oxide and vascular endothelial growth factor (VEGF), further stimulating angiogenesis [15]. Our previous studies demonstrated that Mg-doped CS materials can promote both the osteogenic and angiogenic differentiation of human periodontal ligament cells [15]. In contrast, Sr ions are an essential trace element in the human body that can promote bone formation and inhibit the progression of osteoporosis [16]. Sr shares cellular transport pathways with Ca ions and possesses a strong affinity for binding to the bone matrix during mineralization. Sr exhibits dual-regulatory properties by stimulating osteoblast activity, inhibiting osteoclast activity, reducing bone resorption, and promoting the synthesis and deposition of bone matrix proteins [16]. Sr plays a dual role in promoting and inhibiting bone resorption [17]. Our previous study showed that Sr-doped CS bone cement exhibited enhanced compressive strength, promoting new blood vessel growth and bone regeneration in vivo [18].

Regarding to the fact that angiogenesis and osteogenesis must occur in a coordinated manner, the co-doping of Sr and Mg into calcium silicate ceramics has garnered attention due to their complementary and potentially synergistic effects, by which Mg²⁺ contributes to early-stage cell proliferation and angiogenesis, whereas Sr²⁺ supports late-stage osteogenic differentiation and matrix mineralization. For instance, Lin et al. demonstrated that Mg/Sr co-doped calcium silicate scaffolds significantly upregulated endothelial cell angiogenesis while concurrently promoting the expression of RUNX2 and osteopontin (OPN) in MSCs, ultimately enhancing neovascularization and bone healing in vivo [19]. Our previous study further validated these synergistic effects, showing that Mg/Sr-doped CS cements achieved enhanced mechanical strength, sustained ion release, and superior induction of both osteogenic and angiogenic markers in human periodontal ligament cells (hPDLs) compared to pristine and single-doped formulations [12]. These findings reinforce the significance of developing MSCS materials as promising candidates for bone tissue regeneration.

**Fig. 1:**
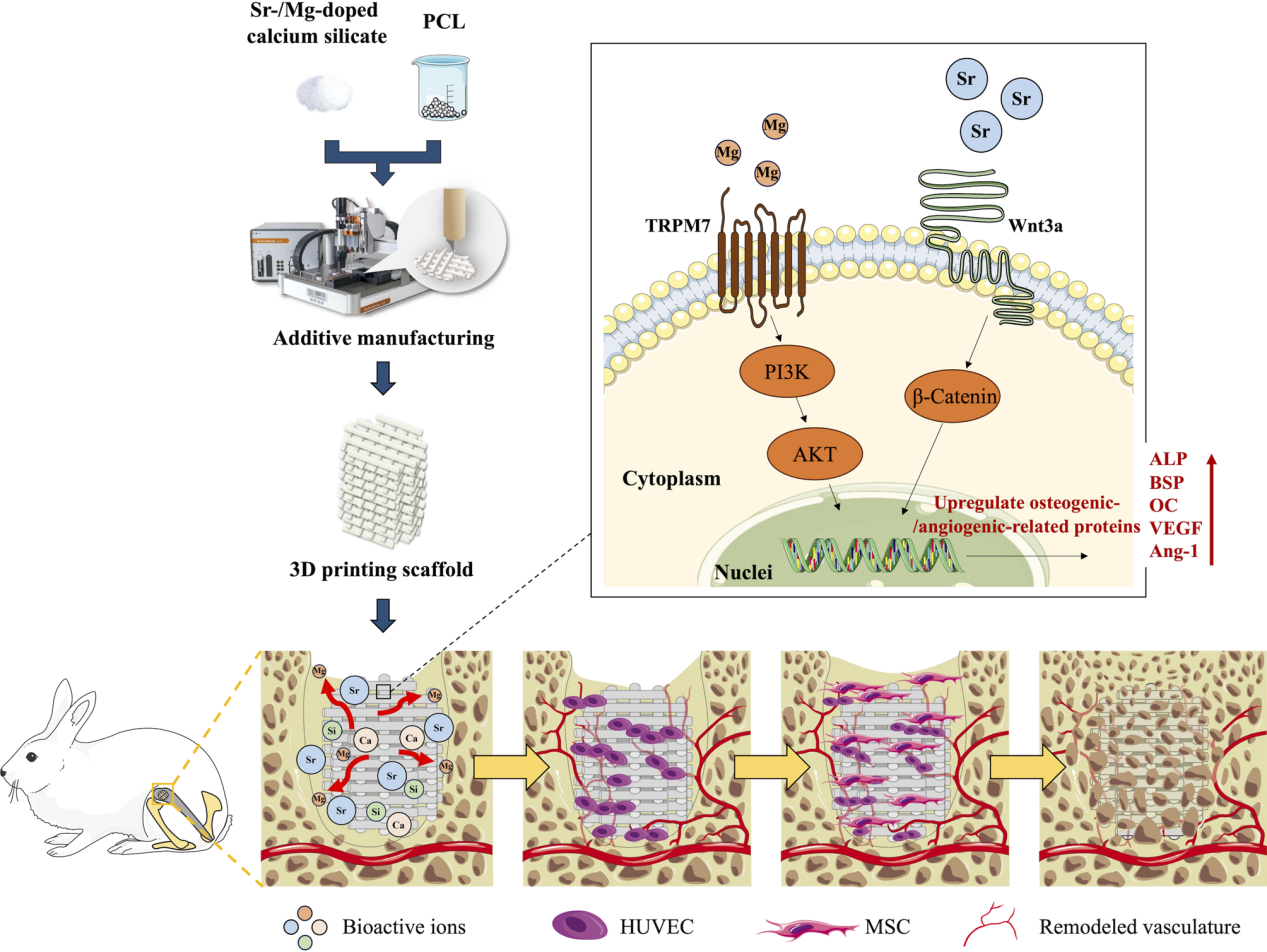
Schematic diagram of the MSCS scaffold and the release of ions affecting Wharton Jelly mesenchymal stromal cell osteogenesis and angiogenesis

A precise scaffold structure design is crucial for the effective application of bioceramics in bone tissue engineering, with parameters such as pore size, interconnectivity, and surface characteristics being of key interest. The traditional methods for fabricating ceramic scaffolds are inadequate for precisely controlling their microstructures. In a previous study, we fabricated various types of ceramic scaffolds using a 3D printing technique that allowed the layer-by-layer deposition of bioceramic paste [20–24]. In this study, we aimed to build on these findings to fabricate Mg/Sr-co-doped CS scaffolds. Specifically, this study focused on the precise control of scaffold architecture and optimization of Mg and Sr doping ratios to maximize therapeutic efficacy. This includes in vitro assessments of cell compatibility and degradation and in vivo evaluation of bone regeneration in a critical-sized bone defect model. The design reported in this study provides a unique strategy to exploit the advantages of Mg and Sr ions, resulting in CS-based scaffolds with enhanced regenerative properties (Fig. 1).

## Materials and methods

### Synthesis of Mg/Sr-doped CS powder

The bioceramics were prepared using a sintering process adapted from previously established methods [12]. High-purity SiO₂, CaO, Al_2_O_3_, MgO, and SrO (Sigma-Aldrich) served as the primary raw materials. The bioceramic formulation was composed of a mixture of CaO, MgO, and SrO (combined to form 65% of the total weight of the mixture), along with 5% Al_2_O_3_ and 30% SiO_2_ by weight. The detailed oxide ratios are outlined in Table 1. To achieve uniform mixing, the raw powders were dispersed in absolute ethanol and ball-milled overnight at a rotational speed of 300 rpm using a Retsch PM100 centrifugal ball mill (Retsch PM100, Germany). The milled mixtures were sintered in a high-temperature furnace, with the temperature gradually raised at a controlled rate of 10℃/min until reaching 1400℃. This temperature was maintained for 2 h to complete the sintering process, after which the furnace was gradually cooled to ambient temperature. The resultant sintered material was milled at 300 rpm for 6 h to obtain a fine powder suitable for further use.

**Table 1:** Composition of the Ca-Si-Mg-Sr powder

Code	CaO	SiO_2_	Al_2_O_3_	MgO	SrO
M0S0	65	30	5	0	0
M0S5	60	30	5	0	5
M5S0	60	30	5	5	0
M5S5	55	30	5	5	5

### 3D printing of Mg/Sr-doped CS scaffolds

To fabricate 3D-printed Mg/Sr-doped CS (MSCS) scaffolds. The Mg/Sr-doped CS powders were prepared as described previously [12]. The doped powders were mixed with 100% ethanol and magnetically stirred at 180 rpm for 2 h to obtain a uniform suspension. The suspension was then subjected to ultrasonic treatment for 30 min to ensure thorough dispersion of the powders. A polymer binder, polycaprolactone (PCL), was incorporated into the mixture until the PCL content reached 50 wt% to produce a printable paste. PCL was first weighed and melted at 180℃ until a transparent liquid was formed. Molten PCL was then gradually added to the MSCS suspension while stirring continuously to ensure homogenous mixing and evaporation of ethanol. The resulting paste was then dried in an oven at 100℃ for 12 h to ensure complete removal of the residual ethanol. The dried paste was loaded into a syringe and extruded using a 3D bioprinter (BioScaffolder 3.1; GeSiM, Grosserkmannsdorf, Germany) to fabricate the scaffolds at a nozzle temperature of 90ºC. Each scaffold layer consisted of seven parallel struts spaced 500 μm apart, and successive layers were oriented 90° perpendicular to the previous one to create a well-defined porous structure. The scaffolds were then labeled based on the Mg/Sr doping concentrations, with groups designated as M0S0 (control, no doping), M0S5, M5S0, and M5S5, corresponding to the weight% of the dopants.

### Phase composition and chemical structure of MSCS scaffolds

The phase composition and chemical structure of the Mg/Sr-doped calcium silicate scaffolds were analyzed using an X-ray diffractometer (XRD, Bruker D8 SSS; Bruker Corporation, Karlsruhe, Germany) with scans in the 2θ range of 20° to 50° under operating conditions of 30 kV and 30 mA and a Fourier transform infrared spectroscopy (FTIR; Bomem DA8.3, Hart- man & Braun, Canada) in the diffuse reflectance mode over the wavenumber range from 4000 to 550 cm^–1^, respectively.

### Measurement of scaffold hydrophilicity and porosity

The water contact angle of each scaffold was measured at room temperature. Scaffolds were positioned on a stainless-steel base, and a droplet of distilled water (10 µL) was carefully dispensed onto the scaffold surface using a micropipette. After 30 s, images of the droplets were captured using a CCD camera. The captured images were then analyzed using ImageJ software (National Institutes of Health) to calculate the water contact angle.

### Microscopic morphology and elemental analysis

The surface morphology of the scaffolds was observed using a field-emission scanning electron microscope (FE-SEM; JEOL JSM-7800 F; JEOL Ltd., Tokyo, Japan) operated in the secondary electron imaging mode at an accelerating voltage of 3 kV. Elemental mapping of the scaffold surfaces, including Ca, Si, Mg, and Sr, was conducted using an energy-dispersive spectrometer (EDS) integrated with the FE-SEM, allowing for qualitative and spatial analysis of the elemental distribution.

### Porosity and compressive strength testing

The porosity of the scaffolds was determined using the specific gravity method. All measurements were conducted at room temperature. For each group, six replicate samples (*N* = 6) were used. Initially, the dry mass of each scaffold sample was recorded as m_s_. The samples were then immersed in a known volume of ethanol and allowed to soak until complete infiltration of the pores was ensured. After saturation, the mass of each ethanol-soaked scaffold was measured and recorded as m_1_. The mass of the remaining ethanol along with the specific gravity bottle was recorded as m_2_, and the initial mass of the ethanol and bottle before sample immersion was noted as m_0_. The porosity (%) was calculated using the following equation:

$${\rm{Porosity }} = \;\left( {{{\rm{m}}_1} - {{\rm{m}}_2} - {{\rm{m}}_{\rm{s}}}} \right)\;/\;\left( {{{\rm{m}}_0} - {{\rm{m}}_2}} \right)$$

The compressive strength of the scaffolds was evaluated using a mechanical testing machine (EZ-Test; Shimadzu Corp., Kyoto, Japan). Static compression tests were conducted at a crosshead speed of 1 mm/min. Rectangular specimens with dimensions of 6.5 × 6.5 × 10.2 mm^3^ were prepared for the tests. Stress-strain curves were generated from the measured data for analysis. All the tests were performed according to the ISO 13314:2011 standard, which outlines the procedures for compressive and plateau stress measurements of porous and cellular materials.

### Immersion test for hydroxyapatite formation

The scaffolds were sterilized by immersion in 75% ethanol and exposed to ultraviolet (UV) light. After sterilization, the scaffolds were rinsed thoroughly with sterile deionized water and immersed in simulated body fluid (SBF) at 37℃ for predetermined time intervals of 3, 7, and 14 days. The SBF was prepared according to the standard Kokubo formulation, consisting of 7.9949 g NaCl, 0.2235 g KCl, 0.147 g K₂HPO₄, 0.3528 g NaHCO₃, 0.071 g Na₂SO₄, 0.2775 g CaCl₂, 0.305 g MgCl₂, and 6.057 g tris(hydroxymethyl)aminomethane per 1000 mL of distilled water. The pH was adjusted to 7.4 using HCl [18]. During the immersion period, the SBF was not renewed in order to mimic static body fluid conditions. Following immersion, the scaffolds were carefully removed, rinsed with deionized water, and dehydrated in an incubator at 37℃ for 12 h. The surface morphology and hydroxyapatite formation on the scaffolds were examined using a FE-SEM and XRD.

### Degradation assessment of the scaffolds

The scaffolds were prepared and sterilized as previously described. After sterilization, the scaffolds were immersed in SBF at 37℃ for designated time intervals of 1, 3, and 6 months. At each time point, the scaffolds were retrieved, rinsed with deionized water, and dehydrated in an incubator at 37℃ for 12 h. The dried scaffolds were weighed using an electronic balance to determine the degradation rate based on the percentage weight loss over time.

### Cell culture

Wharton Jelly mesenchymal stromal cells (WJMSCs) were obtained from Invitrogen (Grand Island, NY, USA). The culture medium consisted of mesenchymal stem cell medium (#7501; ScienCell Research Laboratories, Carlsbad, CA, USA) supplemented with 5% fetal bovine serum, 1% mesenchymal stem cell growth supplement, and 1% penicillin/streptomycin. An osteogenic differentiation medium (ScienCell Research Laboratories) was used for the osteogenic differentiation studies. The cells were seeded onto the scaffolds at a density of 2 × 10^5^ cells/mL. Control groups were cultured in standard tissue culture plates. After seeding, scaffolds were incubated at 37℃ in a humidified atmosphere with 5% CO_2_, and the medium was replaced every 2–3 days or as needed based on the culture conditions.

### Analysis of cell proliferation and morphology

At the designated time points, the scaffolds were removed from the culture and rinsed three times with phosphate-buffered saline (PBS). Cell viability was assessed using a PrestoBlue reagent. Briefly, the scaffolds were incubated with the reagent at 37℃ for 30 min, after which 100 µL of the reactant solution was transferred to a 96-well microplate. The absorbance at 570 nm (reference: 600 nm) was measured in triplicate using a spectrophotometer (Infinite Pro M200, Tecan, Männedorf, Switzerland) for quantitative analysis.

For morphological observation, the scaffolds were rinsed with PBS and fixed in 4% paraformaldehyde for 30 min, followed by three additional PBS washes. Cell membranes were permeabilized using 0.1% Triton X-100 for 10 min. Fluorescent staining was conducted with Alexa Fluor™ 488 Phalloidin (1:500, Invitrogen) for 60 min in the dark to visualize the actin cytoskeleton, followed by DAPI staining (50 nM) for 30 min in the dark to label nuclei. The scaffolds were washed with PBS and deionized water and then imaged using a confocal microscope (Leica TCS SP8, Leica Microsystems, Wetzlar, Germany). Image depths were analyzed using built-in software to capture the three-dimensional cell distribution and morphology.

### Angiogenesis assay

To assess the angiogenic potential of the scaffolds, both endothelial tube formation and cytokine secretion assays were performed. For the tube formation assay, scaffold-conditioned medium was first prepared. Briefly, scaffolds were sterilized by immersion in 75% ethanol, rinsed thoroughly with sterile PBS, and then immersed in endothelial cell medium (ScienCell Research Laboratories) at a ratio of 1 g scaffold to 5 mL medium. After 24 h of incubation at 37 °C, the scaffolds were removed, and the conditioned medium was collected. Subsequently, a volume of 10 µL Matrigel^®^ (Corning) was added into each well of a µ-Slide 15 Well 3D plate (ibidi) and cured at 37 °C for 30 min. Human umbilical vein endothelial cells (HUVECs; Sciencell) were seeded onto the Matrigel-coated wells at a density of 10,000 cells per well in 50 µL of the scaffold-conditioned medium. After 6 h of incubation at 37 °C, capillary-like tube formation was observed and photographed under an optical microscope. Quantification of tube networks, including the number of nodes and total tube length, was performed using ImageJ software.

For the evaluation of angiogenic cytokine secretion from HUVECs cultured on different scaffolds, the expression levels of VEGF and angiopoietin-1 (Ang-1) were quantified using enzyme-linked immunosorbent assay (ELISA) kits (Invitrogen) according to the manufacturer’s protocols. The culture medium was collected at day 7 and day 14, and the concentrations of VEGF and Ang-1 were determined by comparing the absorbance values to those of a standard curve. Experiments were conducted in triplicate with six independent samples per group.

### Osteogenesis assay

To evaluate osteogenic differentiation, WJMSCs were seeded onto the scaffolds at a density of 5 × 10^4^ cells/well in 6-well plates. After 24 h of incubation, the culture medium was replaced with an osteogenic differentiation medium (ScienCell Research Laboratories). The cells were cultured for 3, 7, and 14 days, during which alkaline phosphatase (ALP) activity and the expression of bone sialoprotein (BSP) and osteocalcin (OC) were analyzed at specific time points. For the ALP activity assay, cells were lysed in NP40 cell lysis buffer, and the resulting lysates were centrifuged at 6000 rpm for 15 min. ALP activity was then measured using a pNPP ALP assay kit (Bioassay Systems, Biocore, NSW, Australia) following the manufacturer’s protocol. The total protein content was determined using a bicinchoninic acid protein detection kit (Invitrogen). Relative ALP activity was calculated as the ratio of ALP absorbance to the total protein content. For BSP and OC expression, enzyme-linked immunosorbent assay (ELISA) kits (Invitrogen) were used to quantify the protein levels after 7 and 14 days of culture. Protein concentrations were determined by comparing absorbance values with a standard curve. All experiments were performed in triplicate, with six independent samples per group.

### Western blot

To investigate the intracellular signaling pathways involved in the osteogenic and angiogenic responses, Western blot analysis was performed to assess the expression of β-catenin, transient receptor potential melastatin 7 (TRPM7), phosphoinositide 3-kinase (PI3K), and protein kinase B (Akt) in WJMSCs cultured on different scaffold groups. WJMSCs were seeded on MSCS scaffolds and cultured under osteogenic induction conditions for 7 days. After incubation, cells were lysed using RIPA buffer (Thermo Fisher Scientific) supplemented with protease and phosphatase inhibitor cocktails (Gibco). The lysates were collected and centrifuged at 12,000 ×g for 15 min at 4 °C to obtain the total protein. Protein concentrations were quantified using a BCA protein assay kit (Thermo Fisher Scientific). Equal amounts of protein (30 µg) were separated by SDS-PAGE on 10% polyacrylamide gels and transferred onto PVDF membranes (Millipore). Membranes were blocked with 5% bovine serum albumin (BSA) in PBS-T buffer (PBS with 0.1% Tween 20) for 1 h at room temperature and then incubated overnight at 4 °C with primary antibodies against β-catenin (1:1000, Cell Signaling Technology), TRPM7 (1:1000, Abcam), PI3K (1:1000, Cell Signaling Technology), phosphorylated PI3K (p-PI3K; 1:1000, CST), Akt (1:1000, CST), and phosphorylated Akt (p-Akt; 1:1000, CST). β-actin (1:5000, Abcam) was used as a loading control. After washing, the membranes were incubated with HRP-conjugated secondary antibodies (1:3000, CST) for 1 h at room temperature. The immunoreactive bands were visualized using enhanced chemiluminescence (ECL) substrate (Bio-Rad, USA) and imaged using a ChemiDoc™ XRS + imaging system (Bio-Rad, USA). Band intensities were quantified using ImageJ software (NIH, USA), and relative expression levels were normalized to β-actin.

### Mineralization assay

To assess mineralization on the scaffolds, scaffolds cultured with cells were analyzed after 7 and 14 days. After removing the culture medium, the scaffolds were rinsed with deionized water and fixed in 4% paraformaldehyde (Sigma-Aldrich) for 20 min at room temperature. The fixed scaffolds were washed twice with deionized water and stained with 0.5% Alizarin Red S (ARS; Sigma-Aldrich) solution (pH 4.0) for 20 min on an orbital shaker, with gentle agitation. Following staining, the scaffolds were washed thoroughly with deionized water to remove the excess stain.

Calcium deposition on the scaffolds was visualized using a metallographic microscope (BX53; Olympus). The ARS stain was quantified by immersing the scaffolds in a solution containing 20% methanol and 10% acetic acid for 15 min. The resulting solution was transferred to a 96-well plate and the absorbance was measured at 450 nm using a spectrophotometer. Additionally, scaffolds without cells were also stained and analyzed as the day 0 control group.

### Establishment of a critical-sized bone defect model in rabbits

New Zealand male white rabbits weighing approximately 1.8 kg each were obtained from the Animal Experiment Center of China Medical University. The animals were provided *ad libitum* access to food and water and acclimated to their environment for one week prior to experimentation. Anesthesia was administered using a gas anesthesia machine, and the rabbits were maintained under continuous anesthesia with 5% isoflurane in oxygen during the surgical procedure. After shaving and disinfecting the hind legs, an incision was made along the thigh and extended toward the inner lower leg to expose the lateral femoral condyle. A critical-sized bone defect was created using a dental handpiece to drill a hole with a diameter of 7 mm and depth of 8 mm in the femoral condyle. The defect site was irrigated with PBS to remove debris, and MSCS scaffolds (6.5 mm diameter and 7.8 mm height) were implanted into the defect. The wound was then sutured and an anti-inflammatory ointment was applied along the incision. Finally, the rabbits were sacrificed via CO_2_ asphyxiation at 4 and 8 weeks post-implantation for further analysis.

### Micro-computed tomography (µ-CT) and histological analysis

The scaffold-implanted regions were scanned using a multi-scale X-ray nano-CT system (SkyScan 2211, Bruker, Belgium) with parameters set at 100 kVp, 330 µA, and 20 W output. The images were reconstructed using Insta Recon software (Bruker, Belgium), and the bone volume (BV/TV) and trabecular thickness (Tb.Th) were analyzed with Avizo 8.0 software (Visualization Sciences Group, France). Following µ-CT imaging, sections of the bone defects were prepared using the freeze-section technique. The sections were stained with hematoxylin-eosin (HE) for general tissue morphology, Masson’s trichrome (MT) for collagen fiber visualization, and Von Kossa (VK) staining for calcified bone. All samples were observed and analyzed using an optical microscope.

### Statistical analysis

The data were analyzed using one-way analysis of variance to evaluate differences between experimental groups. Scheffe’s multiple comparison test was performed to determine significant deviations among the samples. A p-value < 0.05 was considered statistically significant, with significant differences marked by “*” or “#” in the group comparisons.

## Results and discussion

### Phase composition and chemical structure of the MSCS/PCL composites

**Fig. 2:**
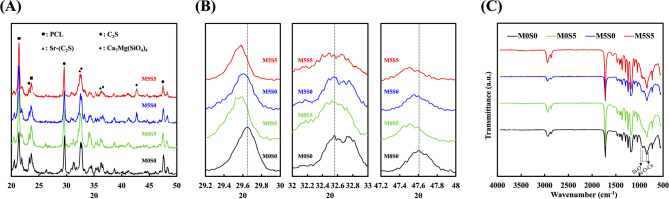
(**A**) XRD patterns, (**B**) enlarged XRD patterns highlighting selected 2θ regions, and (**C**) Fourier transform infrared (FTIR) spectra of MSCS scaffolds with different compositions (M0S0, M0S5, M5S0, and M5S5)

XRD analysis was conducted to evaluate the phase composition of the PCL composites with different doping compositions, including undoped calcium silicate (M0S0), Sr-doped calcium silicate (M0S5), Mg-doped calcium silicate (M5S0), and Sr/Mg-co-doped calcium silicate (M5S5). The resulting diffraction patterns are shown in Fig. 2A. The diffraction peak corresponding to PCL was observed at a 2θ of approximately 21.5° in all patterns, confirming its presence in the composite. The results indicated that the M0S0 group exhibited characteristic diffraction peaks that align well with standard dicalcium silicate (Ca_2_SiO_4_) patterns [25]. The major peaks were observed at approximately 2θ = 23.6°, 29.6°, 32.6°, and 47.6°, which were consistent with β-dicalcium silicate (C2S), a known bioactive phase commonly found in calcium silicate-based materials. For the M0S5 group, the diffraction pattern retained the primary peaks associated with dicalcium silicate; however, slight shifts in the peak positions were observed. As seen in Fig. 2B the most noticeable shifts occurred at 2θ = 29.6°, 32.6°, and 47.6° where the peaks appeared slightly displaced toward lower angles. This shift suggests an increase in lattice spacing, which is expected due to the incorporation of Sr^2+^ ions, which have a larger ionic radius than Ca^2+^ [26]. Despite these shifts, no significant secondary crystalline phases related to Sr compounds were detected, indicating that Sr was effectively incorporated into the dicalcium silicate lattice without forming separate Sr-based phases. For the M5S0 sample, the XRD pattern showed the presence of new peaks at 2θ = 33.4°, 36.8°, and 42.8°, which correspond to the recognized bioactive phase in magnesium-doped silicates (bredigite, Ca_7_Mg(SiO_4_)_4_) [12]. These additional peaks suggest that Mg was successfully integrated into the silicate structure, leading to partial phase transformation from β-C_2_S to Ca_7_Mg(SiO_4_)_4_. Compared to M0S0 and M0S5, the peak intensities in M5S0 appeared broader, which may indicate a reduction in crystallinity. Additionally, some minor peak shifts toward higher angles were observed, which was expected due to the smaller ionic radius of Mg^2+^ compared to Ca^2+^, resulting in lattice contraction [27]. Additionally, the M5S5 group exhibited a diffraction pattern that integrates the features observed for M0S5 and M5S0. The characteristic peaks of β-C_2_S were still present but appeared slightly broadened, suggesting partial disruption of the original crystalline structure. Peaks associated with Ca_7_Mg(SiO_4_)_4_ remain visible, indicating that Mg incorporation was maintained. However, compared to M5S0, the intensity of the Ca_7_Mg(SiO_4_)_4_ peaks appeared slightly reduced, suggesting a complex interaction between Sr and Mg within the crystal lattice. The observed shifts in the peak positions suggest that Sr and Mg co-doping modifies the overall crystalline structure of the CS ceramic, potentially leading to changes in its mechanical properties, bioactivity, and ion release behavior.

The chemical structures of the composites with different dopants were examined using Fourier transform infrared spectrometry (FTIR). As shown in Fig. 2C, all groups exhibited characteristic absorption bands, indicating that Sr and Mg doping did not significantly alter the fundamental functional groups within the PCL matrix or the calcium silicate structure. All composites showed typical PCL peaks at 2947 and 2865 cm⁻¹, 1720 cm⁻¹, 1171 cm⁻¹, and 1100 cm⁻¹ which were attributed to stretching vibrations of CH_2_, C = O, and C–O–C, C–O, respectively [26]. Furthermore, the peaks in the 1000–800 cm⁻¹ region were attributed to Si–O bending vibrations, while the bands at 500–600 cm⁻¹ correspond to Si–O–Ca vibrations, confirming the presence of calcium silicate [28]. These features remained consistent among all the groups, indicating that Sr and Mg doping did not cause major alterations in the chemical structure of calcium silicate. Collectively, these data suggest that the doping process primarily affects the crystalline and phase compositions rather than changes the structure of the primary silicate network, as evidenced by the consistent Si–O and Si–O–Ca peaks.

### Surface wettability of the MSCS scaffolds

**Fig. 3:**
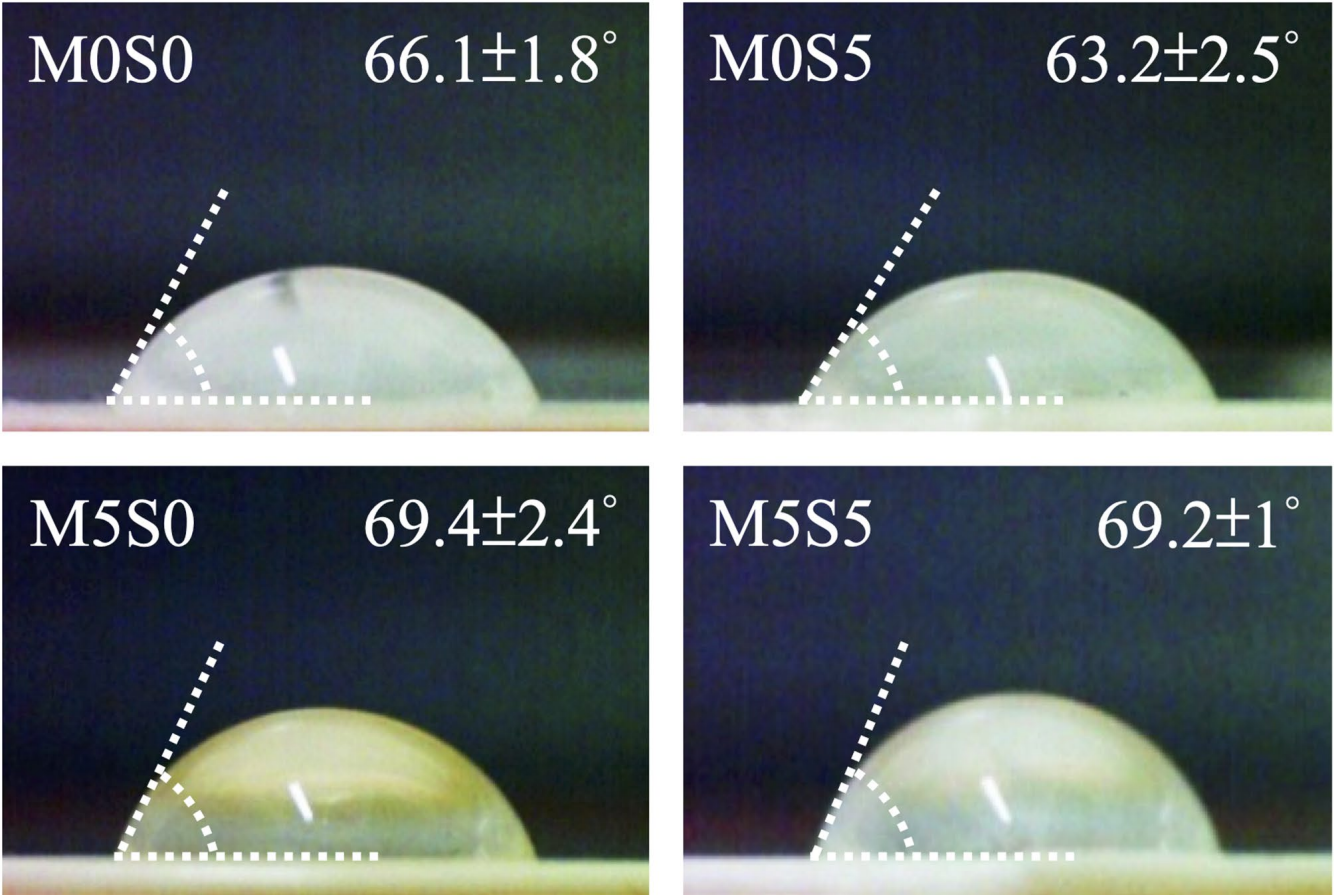
Water contact angle measurements of MSCS scaffolds with different compositions

Owing to its poor wettability, PCL alone does not provide an ideal surface for cell attachment and proliferation. Hydrophobic surfaces limit protein adsorption, which is essential for initial cell adhesion, potentially delaying early-stage cellular interactions. In addition, limited water penetration into the scaffold can lead to a slow degradation rate, which may hinder timely scaffold resorption and subsequent tissue integration. Furthermore, hydrophobic surfaces have been associated with increased bacterial adhesion, raising concerns regarding the risk of implant-associated infections [29]. These drawbacks highlight the need for surface modification or material blending strategies to enhance the bioactivity and wettability of PCL-based scaffolds. In this study, the wettability of the 3D-printed composite scaffolds was evaluated using water contact angle (WCA) measurements. As shown in Fig. 3, the results demonstrated that the WCA values of the different scaffolds ranged between 60° and 70°, which were lower than the PCL surface values of over 80° reported in the literature [30]. This finding indicates that incorporating CS-based bioceramics into the PCL matrix significantly improved the surface wettability of the scaffold. Compared with M0S0, a slight reduction in the contact angle was observed for M0S5, suggesting increased hydrophilicity. However, the opposite trend was observed for the Mg-doped CS group, where the contact angle increased slightly. Although the exact reason for these variations remains unclear, it was postulated that the WCA value was significantly influenced by the physicochemical characteristics of the incorporated CS particles, including their size, shape, hydrophilicity, crystallinity, and surface exposure level. Nevertheless, all composite scaffolds exhibited acceptable hydrophilicity, which may be attributed to the presence of silanol (Si–OH) functional groups in CS. Consequently, the improved wettability of the scaffolds is expected to provide a more favorable microenvironment for cell adhesion and proliferation, which are essential for bone tissue engineering applications. In addition to improving cell-material interactions, the enhanced wettability of the scaffolds may also play a role in optimizing scaffold degradation. A more hydrophilic surface allows better water infiltration, which promotes the hydrolytic breakdown of PCL and ensures a more controlled degradation profile [31].

### Morphological and elemental composition of MSCS scaffolds

**Fig. 4:**
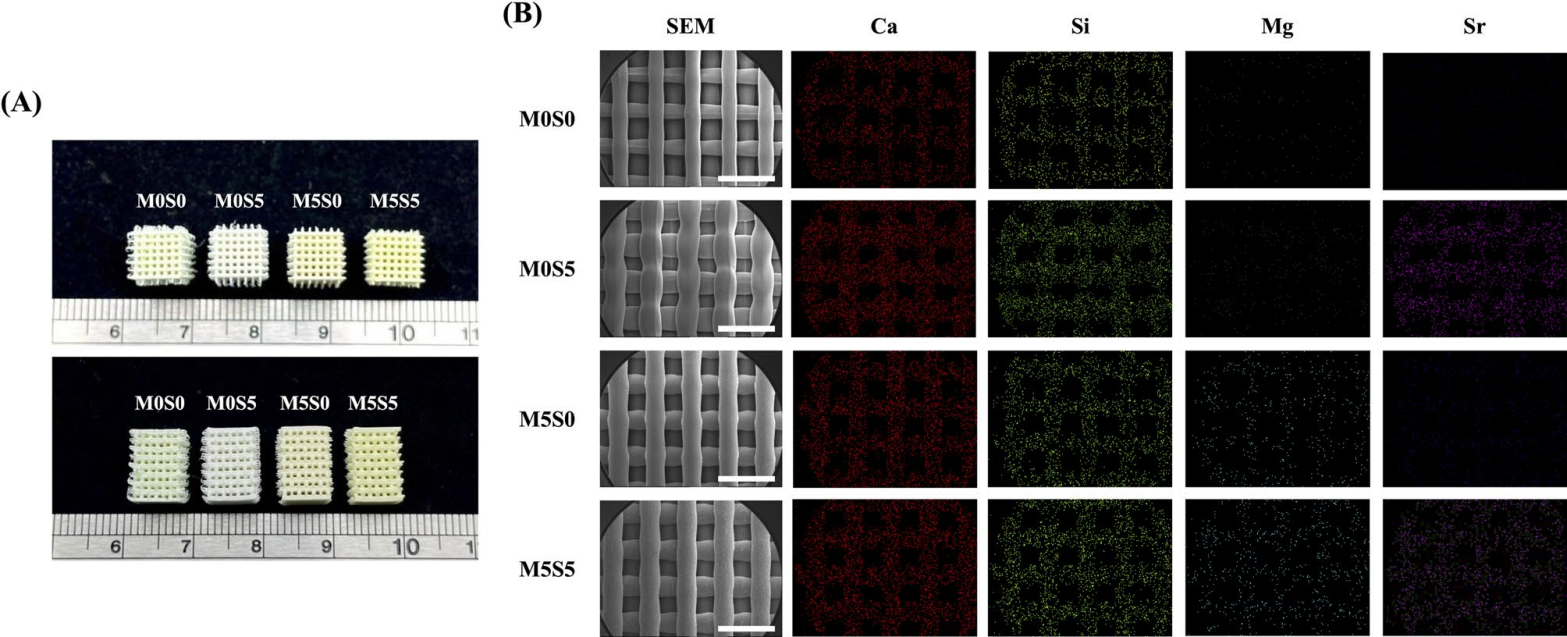
(**A**) Optical images of the MSCS scaffolds with different compositions. (**B**) Scanning electron microscopy images and energy-dispersive spectrometry element mapping of calcium (Ca), silicon (Si), magnesium (Mg), and strontium (Sr) in the MSCS scaffolds. Scale bar: 1.4 mm

Photographic images (Fig. 4A) of the different scaffolds revealed that all groups exhibited well-defined macroporous structures, demonstrating the successful fabrication of the composite scaffolds via 3D printing. Specifically, the scaffolds maintained their structural integrity without visible defects or distortions, indicating their good printability and mechanical stability [32]. Differences in the color and surface texture between groups suggested variations in composition, which were further analyzed using SEM and EDS. The SEM micrographs (Fig. 4B) provided a detailed visualization of the microstructure of the scaffolds. All the scaffolds exhibited a smooth surface with a well-preserved porous structure. EDS elemental mapping confirmed the successful incorporation of Sr and Mg into the scaffolds. In the M0S0 scaffold, the elemental distribution primarily comprised Ca and Si. In the M0S5 scaffold, Sr was clearly detected and was evenly distributed across the scaffold matrix, suggesting that Sr was successfully incorporated into the silicate structure without significant phase separation. Similarly, the M5S0 scaffold exhibited the distinct presence of Mg with a uniform distribution. The M5S5 scaffold contained both Sr and Mg with a homogenous spatial distribution, indicating effective co-doping into the scaffold structure. Collectively, the elemental mapping results confirmed the successful incorporation of Sr and Mg, which are known to enhance bioactivity and osteogenic properties. Sr is known to stimulate osteoblast activity and inhibit osteoclast-mediated bone resorption, contributing to improved bone regeneration [33]. Mg, in contrast, plays a key role in enhancing angiogenesis and influencing bone mineralization pathways [34]. The homogeneous distribution of Sr and Mg in the M5S5 scaffold suggests that co-doping does not lead to phase segregation, which is beneficial for maintaining material uniformity and consistent ion release. Further in vitro and in vivo studies are necessary to evaluate the biological responses of these composite scaffolds and determine the optimal doping levels for enhanced osteogenic performance.

### Porosity and mechanical properties of the MSCS scaffolds

**Fig. 5:**
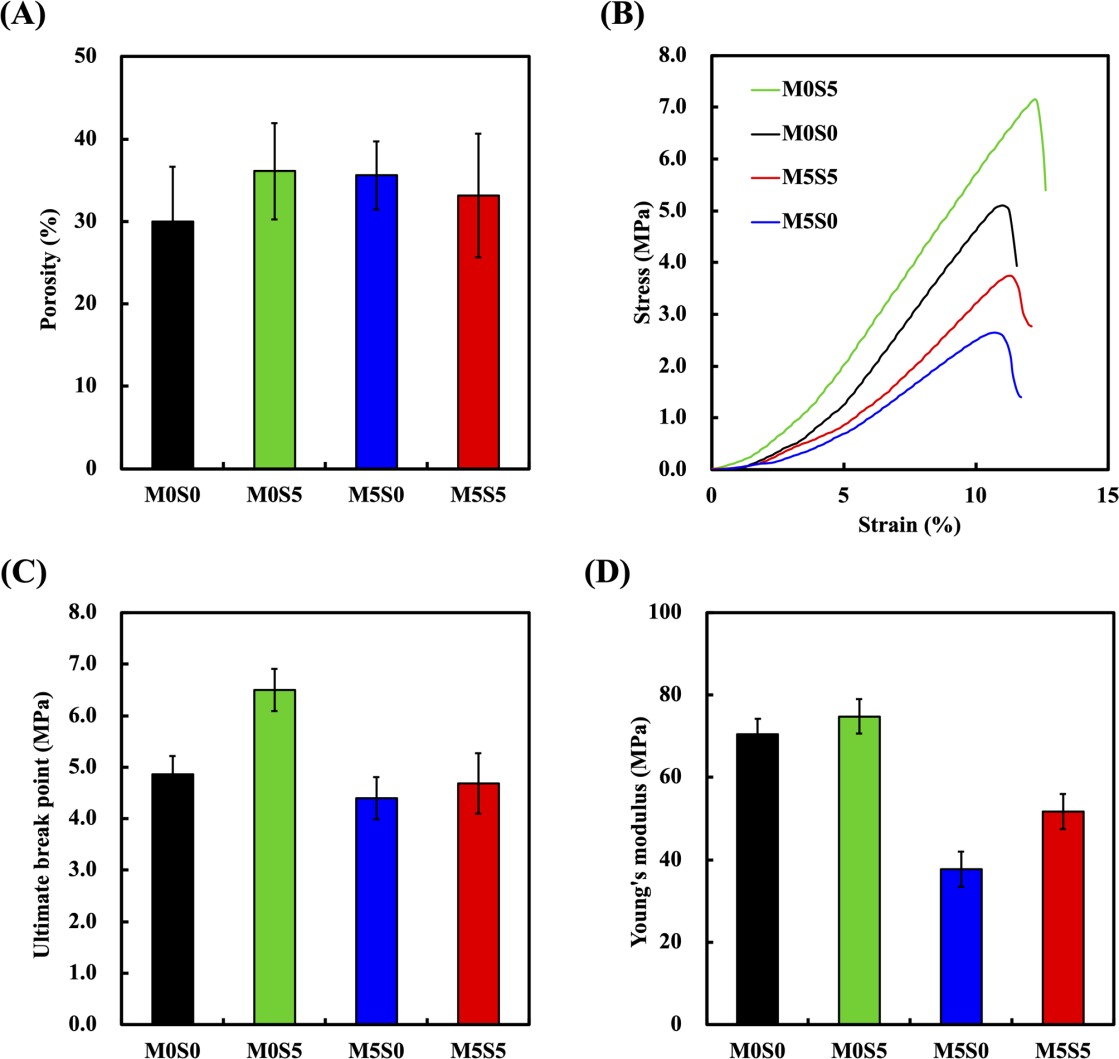
(**A**) Porosity, (**B**) stress-strain curves, (**C**) ultimate break point, and (**D**) Young’s modulus of the MSCS scaffolds with different compositions. Data are presented as the means ± standard error of the mean; *n* = 6 for each group

The porosity of the scaffolds was assessed using the specific gravity method, and the results showed that the M0S0, M5S0, M0S5, and M5S5 groups exhibited average porosities of 30.0 ± 6.7%, 36.1 ± 5.8%, 35.6 ± 4.1%, and 33.2 ± 7.5%, respectively (Fig. 5A). These values suggest a comparable level of macroporosity among the different groups, with no statistically significant differences observed (*p* > 0.05), confirming the reproducibility of the 3D printing process across different doping compositions. The mechanical properties of the 3D-printed composite scaffolds were evaluated to assess their ability to withstand mechanical loads in bone tissue engineering applications and provide insights into the effects of Sr and Mg doping on scaffold performance. The compressive strength and modulus results for the 3D-printed composite scaffolds (Fig. 5B–D) revealed that incorporating Sr- and Mg-doped CS ceramics into the PCL matrix significantly influenced the mechanical performance of the scaffolds. The compressive moduli of the scaffolds for M0S0, M0S5, M5S0, and M5S5 were 63.5, 67.4, 34.0, and 46.5 MPa, respectively, while the corresponding compressive strengths were 4.9, 6.5, 4.4 and 4.7 MPa, respectively. Compared with M0S0, the incorporation of Sr (M0S5) significantly increased the compressive modulus and improved the compressive strength. This enhancement is attributed to the ability of Sr to stabilize the calcium silicate lattice and strengthen the interfacial bonding within the composite matrix [12]. In contrast, incorporating Mg into the M5S0 scaffold substantially reduced both modulus and strength compared with those of M0S5. This decline is likely due to the smaller ionic radius of Mg, which introduces lattice disorder and disrupts the cohesive structure of the material, leading to a less coordinated structure and weakening of its mechanical strength [35]. As expected, the co-doped scaffold (M5S5) exhibited an intermediate performance between M0S5 and M5S0. Although their mechanical properties are better than those of M5S0, they are still inferior to those of M0S5. This result suggests a tradeoff between the stabilizing effects of Sr and the weakening effects of Mg. The simultaneous presence of Sr and Mg appeared to balance their individual effects but did not entirely counteract the structural disruptions caused by Mg doping. Besides, incorporating dopants may alter interatomic bonding and introduce microstructural heterogeneities, such as secondary phases (e.g., Ca_7_Mg(SiO_4_)_4_), which influence the mechanical response under compression. Although the M5S0 and M5S5 scaffolds exhibited reduced mechanical properties compared to the undoped M0S0 scaffold, the compressive moduli and strengths of all scaffolds remained within the range of cancellous bone (4–12 MPa for compressive strength and 100–500 MPa for compressive modulus) [36]. This indicates their potential suitability for non-load-bearing or low-load-bearing bone regeneration applications. Future optimization of scaffold design, such as adjusting porosity and incorporating advanced architectural patterns, could further enhance the mechanical strength of the scaffolds [37].

### Bioactivity and degradation behavior of MSCS scaffolds

**Fig. 6:**
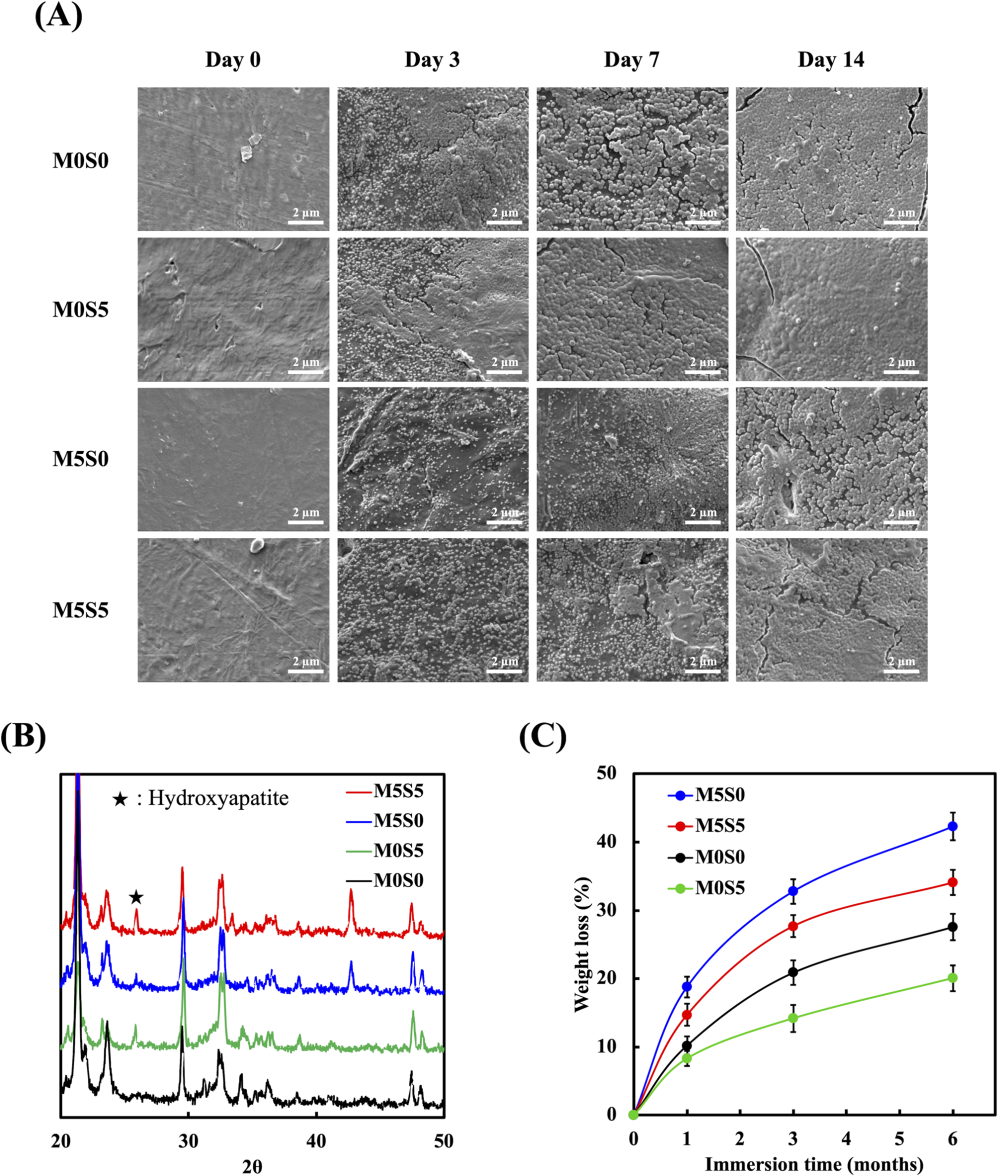
(**A**) Scanning electron microscopy images of MSCS scaffolds after immersion in SBF for 0, 3, 7, and 14 days. Scale bar: 2 μm. (**B**) Weight loss profiles of the MSCS scaffolds over a 6-month immersion period in SBF. Data are presented as the means ± standard error of the mean; *n* = 6 for each group

The bioactivity and degradation behavior of the 3D-printed composite scaffolds, which are critical for assessing their suitability for bone tissue engineering applications, were evaluated by immersion in SBF for various durations. The SEM images (Fig. 6A) show the surface morphology of the scaffolds before and after immersion in SBF. Before immersion, all scaffolds exhibited a relatively smooth surface with slightly wrinkled textures, characteristic of a semi-crystalline polymer surface topology, implying that the incorporated CS-based particles were buried in the PCL matrix. After immersion in SBF, noticeable morphological alterations were observed in all groups. The formation of apatite-like granules became evident on the scaffold surfaces, with the deposition amount increasing over prolonged immersion periods despite the limited exposure of the incorporated CS. Based on the XRD patterns in Fig. 6B, single- or co-doped CS scaffolds showed the emergence of characteristic peaks at approximately 2θ = 26.2º, corresponding to the (002) planes of hydroxyapatite, confirming mineral formation upon SBF immersion [38]. Among all groups, the M0S0 scaffold exhibited excellent apatite-forming ability to facilitate the formation of a dense and continuous mineral layer across the surface over time, which is in good accordance with our previous findings [18, 39]. M0S5 displayed an even more pronounced apatite-forming ability, with the apatite granules forming a compact and homogenous layer within a shorter immersion period than M0S0, suggesting improved bioactivity. However, the apatite-like granules deposited on the M5S0 surface were less densely packed and more heterogeneously distributed than those deposited on the M0S0 surface. Moreover, the M5S5 scaffold achieved a balance between mineralization enhancement and structural uniformity, forming a more continuous apatite layer than the M5S0 scaffold but with a slightly lower density than the M0S5 scaffold. These results indicate that the interaction between Sr and Mg affects apatite nucleation and growth, leading to the formation of a bioactive surface with a moderate mineralization potential.

Next, the degradation profiles of the scaffolds were obtained using weight loss measurements after immersion in SBF. As shown in Fig. 6C, all scaffolds exhibited a burst increase in weight loss at varying rates depending on the composition of the CS dopants during the first month of immersion, followed by a gradually declining degradation rate at longer immersion periods. After one month, the M0S0 scaffold exhibited a weight loss of 10.3%, whereas the M0S5 scaffold showed a slightly lower weight loss of 8.4%, suggesting that Sr incorporation effectively moderated the initial degradation rate. The M5S0 scaffold exhibited the highest weight loss (18.9%). In contrast, the M5S5 scaffold demonstrated a weight loss of 14.8%, suggesting that the combined effect of Sr and Mg provided a more tunable degradation profile than the samples of other compositions. As the immersion period progressed, the degradation rates gradually decreased, indicating that the initial burst release of ions and structural dissolution stabilized over time. By six months, the weight loss percentages further increased to 27.6%, 20.1%, 42.2%, and 34.0% for the M0S0, M0S5, M5S0, and M5S5 scaffolds, respectively. These results demonstrate that the degradation rate of scaffolds immersed in SBF is highly dependent on the ion substitution in CS. This further altered the leaching kinetics of Ca^2+^ and soluble silica species, local ionic supersaturation, and the integrity of the silicate network from the CS matrix [40]. Specifically, the Sr-doped scaffolds (M0S5) exhibited a controlled degradation rate, facilitating stable Ca^2+^ release and enhancing apatite formation. However, Mg doping (M5S0) accelerated scaffold degradation, causing rapid breakdown and impairing apatite layer formation due to excessive ion leaching. In contrast, the co-doped Sr/Mg scaffold (M5S5) exhibited balanced degradation and mineralization, mitigating the excessive solubility of Mg while maintaining its bioactivity.

### Cell proliferation and cytoskeletal organization on the MSCS scaffolds

**Fig. 7:**
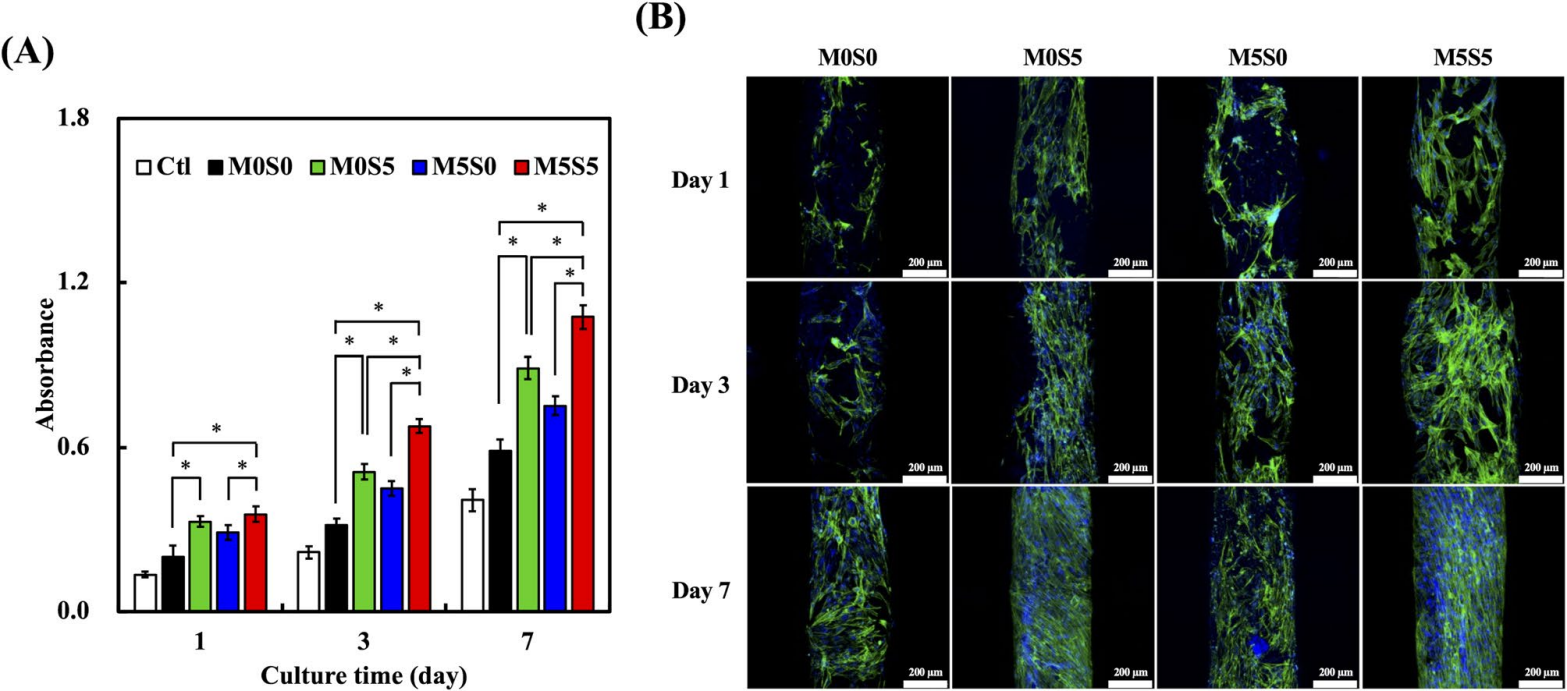
(**A**) Cell proliferation of WJMSCs on the MSCS scaffolds at days 1, 3, and 7. Data are presented as the means ± standard error the mean; *n* = 6 for each group. The symbol “*” denotes statistically significant differences (*p* < 0.05) between the groups. (**B**) Fluorescence images of WJMSCs on the MSCS scaffolds at days 1, 3, and 7. F-actin is stained green, while nuclei are stained blue. Scale bar: 200 μm

**Fig. 8:**
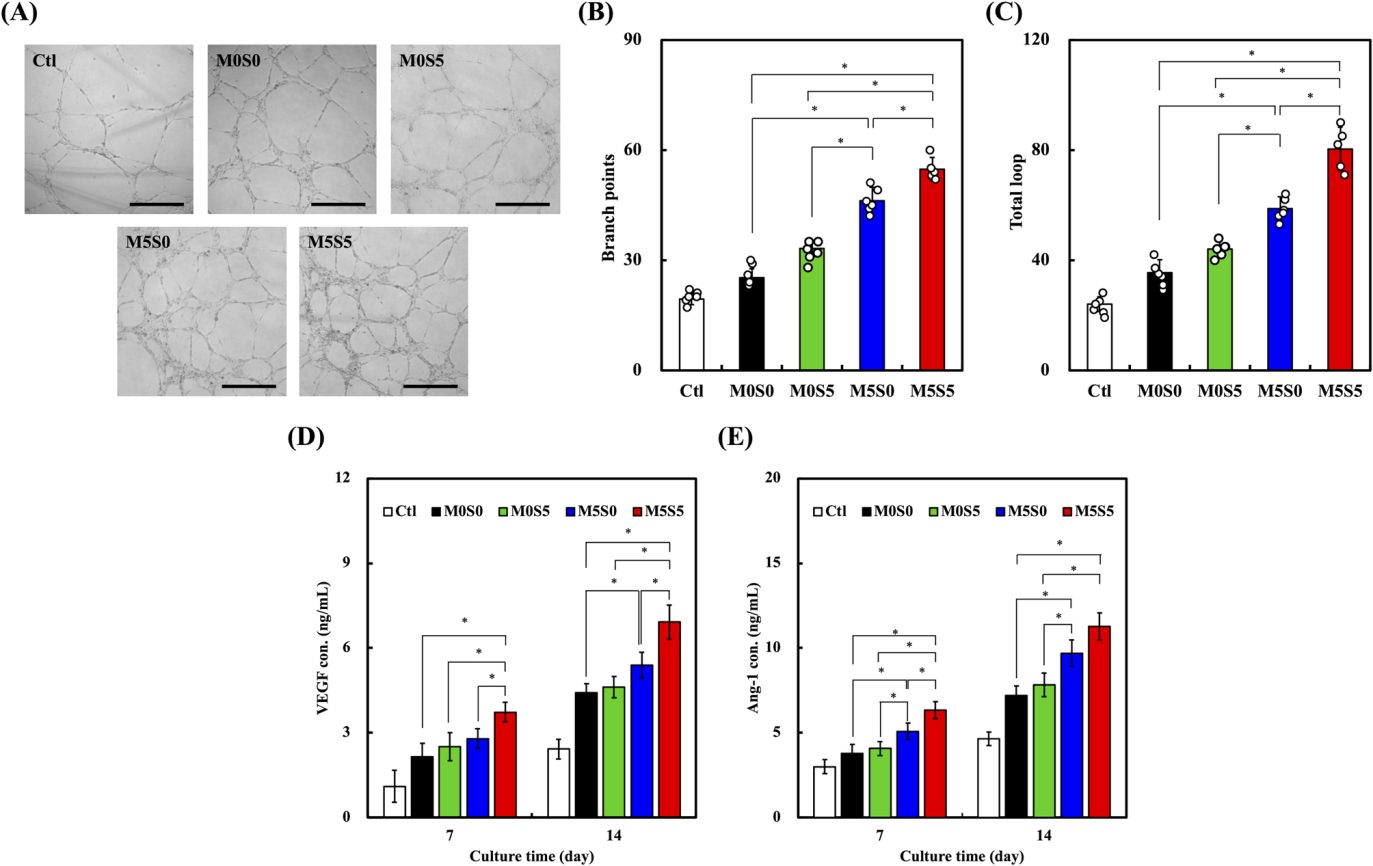
(**A**) Tube formation assay of HUVECs cultured with conditioned media from MSCS scaffolds of varying compositions. Scale bar: 500 μm. (**B**) Quantification of branch points and (**C**) total loops formed by HUVECs. (**D**) VEGF and (**E**) Ang-1 secretion levels on days 7 and 14. Data are presented as the means ± standard error of the mean; *n* = 6 for each group. The symbol “*” denotes statistically significant differences (*p* < 0.05) between the groups

A PrestoBlue assay was performed to assess the proliferation of WJMSCs cultured on the MSCS scaffolds, and the absorbance values are presented in Fig. 7A. The control group (Ctl) comprised cells cultured on standard tissue culture plates without scaffolds. Compared with the Ctl group, all scaffold groups (M0S0, M0S5, M5S0, and M5S5) exhibited significantly higher proliferation rates on days 3 and 7 (*p* < 0.05), suggesting that the CS-based scaffolds provided a favorable environment for various types cells growth [41]. Among the scaffold groups, the Sr-containing scaffolds (M0S5 and M5S5) showed a more pronounced increase in cell proliferation than M5S0 and M0S0, particularly on days 3 and 7. On day 1, the proliferation rate in the Sr-treated groups (M0S5 and M5S5) was higher than that in the M5S0 and M0S0. This trend continued over time, with M0S5 and M5S5 maintaining significantly higher absorbance values on days 3 and 7. Notably, the M5S5 group demonstrated the highest proliferation rate, exceeding that of the M0S5 group by 23% on day 3 and 17% on day 7. These findings indicate that combining Mg and Sr into CS scaffolds synergistically enhances WJMSC proliferation while maintaining cytocompatibility. Previous studies suggested that Mg promotes early cell adhesion and proliferation, whereas Sr enhances osteogenic differentiation by stimulating extracellular matrix production and bone-specific protein expression (Fig. 8).

Fluorescence staining corroborated the proliferation assay results (Fig. 7B). The results of the cytoskeletal (green, Alexa Fluor™ 488 Phalloidin) and nuclear (blue, DAPI) staining demonstrated well-spread cell attachment on all scaffolds. Specifically, the cells cultured on the M0S5 and M5S5 scaffolds exhibited a more organized and elongated morphology with higher confluence than those cultured on the M5S0 scaffold, particularly on days 3 and 7. By day 7, the M5S5 scaffolds were almost entirely covered with spindle-shaped WJMSCs, indicating enhanced cell attachment and proliferation [42]. In contrast, the M5S0 group showed a comparatively lower cell density, consistent with the PrestoBlue assay results. Taken together, these results suggested that Sr incorporation enhanced the proliferation and cytoskeletal organization of WJMSCs, particularly when combined with Mg-doped CS scaffolds. The synergistic effects of Mg and Sr may contribute to the improved bioactivity and cellular responses observed in the Mg and Sr co-doped scaffolds.

### Angiogenic potential of HUVECs cultured with extracts from MSCS scaffolds

Angiogenesis is essential for bone regeneration because it facilitates vascularization and nutrient transport to support newly formed bone tissue. To evaluate the angiogenic potential of the MSCS scaffolds, HUVECs were cultured in conditioned media containing the scaffold extracts, and their tube formation ability was assessed (Fig. 8A). The results showed that HUVECs exposed to the M5S5 scaffold extract formed the most extensive and interconnected capillary-like structures, followed by those exposed to the M5S0 and M0S5 extracts. In contrast, cells cultured with extracts from the M0S0 and control (Ctl) groups exhibited significantly fewer branch points and less organized networks. Quantification of the branch points (Fig. 8B) and total loops (Fig. 8C) confirmed that the M5S5 scaffold extract significantly enhanced endothelial network formation compared with extracts from the other groups (*p* < 0.05). Although the M5S0 and M0S5 scaffold extracts also promoted tube formation, their angiogenic capacities remained lower than that of M5S5, indicating that co-doping with Sr and Mg provided the most favorable conditions for endothelial cell networking. In previous studies, the biological benefits of CS-based ceramics have been closely associated with their ionic dissolution products (e.g., Ca^2+^ and Si^4+^), which play pivotal roles in modulating cellular behaviors [9, 12, 23]. Notably, Si⁴⁺ ions have been associated with enhanced collagen synthesis, extracellular matrix (ECM) deposition, and increased cell proliferation. These cellular responses contribute to a favorable microenvironment that promotes the secretion of pro-angiogenic factors such as VEGF, FGF-2, and hepatocyte growth factor (HGF). Such ion-induced effects are mediated through the activation of intracellular signaling cascades including PI3K/Akt and MAPK pathways, underscoring the therapeutic potential of silicon-rich ceramics in vascularized tissue regeneration [43].

**Fig. 9:**
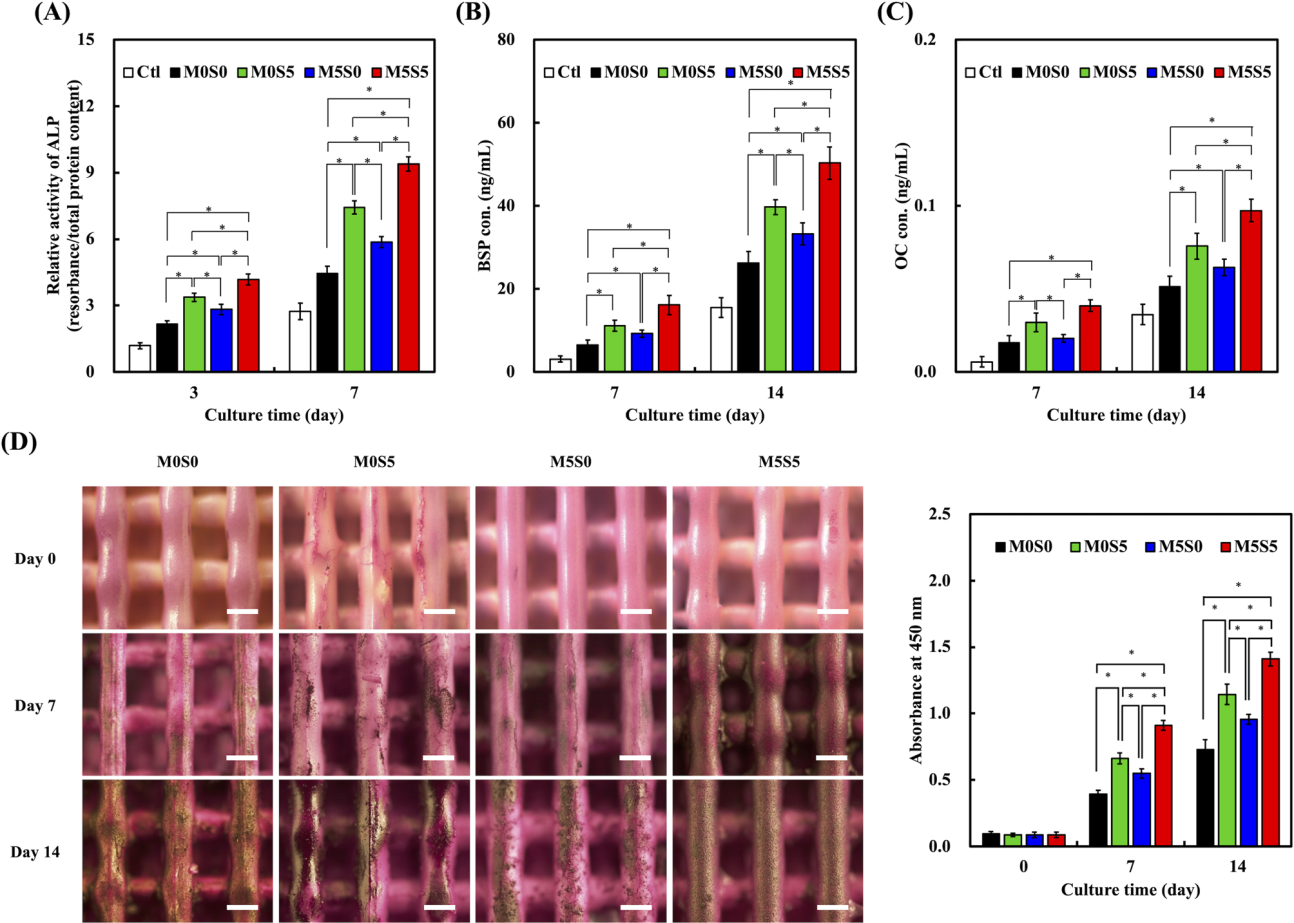
(**A**) Alkaline phosphatase activity, (**B**) bone sialoprotein concentration, and (**C**) osteocalcin concentrations in Wharton Jelly mesenchymal stromal cells cultured on the MSCS scaffolds at days 3, 7, and 14. (**D**) Alizarin Red S staining images of mineralized matrix deposition at days 7 and 14 and the corresponding quantification of absorbance at 450 nm. Data are presented as the means ± standard error of the mean; *n* = 6 for each group. The symbol “*” denotes statistically significant differences (*p* < 0.05) between the groups

To further investigate the molecular basis of this enhanced angiogenesis, ELISA was performed to quantify the secretion of VEGF and Ang-1 on days 7 and 14 (Fig. 8D and E). Both VEGF and Ang-1 levels were significantly higher in the Sr- and Mg-containing groups (M0S5, M5S0, and M5S5) than in the M0S0 and control groups (*p* < 0.05), with M5S5 inducing the highest secretion levels. VEGF is a key regulator of angiogenesis, facilitating endothelial cell proliferation and migration, while Ang-1 contributes to stabilizing newly formed blood vessels. The presence of Sr in the M0S5 and M5S5 groups contributed to an early increase in VEGF secretion, whereas the combination of Sr and Mg in M5S5 resulted in sustained upregulation of both VEGF and Ang-1 secretion, further enhancing angiogenic activity. The observed angiogenic effects aligned with the ion release profiles of the scaffolds, suggesting that Sr and Mg exert complementary effects on endothelial activation and vessel formation. Sr ions have been reported to stimulate VEGF expression via the PI3K/Akt signaling pathway, promoting endothelial cell proliferation and migration [44]. Meanwhile, magnesium ions enhance endothelial cell survival and function by regulating nitric oxide production, which plays a critical role in vascular homeostasis [45]. Therefore, the co-doping of Sr and Mg in M5S5 may generate a synergistic effect, not only enhancing initial endothelial tube formation but also maintaining a pro-angiogenic microenvironment through sustained angiogenic factor secretion. These results suggest that the Mg/Sr co-doped CS scaffolds provide an optimized ionic microenvironment that facilitates vascularization, which is crucial for improving scaffold integration and supporting bone tissue regeneration.

### Osteogenic differentiation and mineralization

The osteogenic potential of the Mg/Sr-doped CS scaffolds was evaluated based on ALP activity, BSP and OC secretion, and calcium deposition. ALP activity (Fig. 9A), an early marker of osteogenic differentiation, progressively increased across all groups, with M5S5 consistently displaying the highest activity. A significant enhancement was observed for M5S5 compared to the other compositions on days 3 and 7 (*p* < 0.05), suggesting that the presence of both Sr and Mg facilitated early-stage osteoblast differentiation. The incorporation of Mg may contribute to the regulation of osteogenic signaling pathways, further amplifying ALP activity. In contrast, the M5S0 scaffold exhibited lower ALP activity, likely because of excessive ion release, disrupting cellular homeostasis. Similarly, on days 7 and 14, BSP secretion (Fig. 9B), a key regulator of extracellular matrix mineralization, was significantly higher in cells grown on M5S5 scaffolds (*p* < 0.05), confirming its role in promoting bone matrix formation. OC expression (Fig. 9C), a late-stage osteogenic marker, showed no significant differences on day 7, but was markedly elevated in cells grown on the M5S5 scaffold on day 14 (*p* < 0.05), underscoring the sustained osteogenic effect of Sr. Although cells cultured with the M0S5 scaffold also exhibited increased BSP and OC expression, indicating a positive role of Sr in bone matrix formation, its effect was slightly lower than that of M5S5, reinforcing the importance of Mg in enhancing matrix mineralization. In contrast, M5S0 displayed significantly lower BSP and OC levels, suggesting that its rapid degradation compromised the scaffold stability and disrupted the microenvironment necessary for late-stage osteogenesis [46, 47].

Calcium deposition, a hallmark of bone matrix mineralization, was assessed using ARS staining and quantitative calcium content analysis (Fig. 9D). On day 0, no visible mineralization was observed across all groups. However, by day 7, calcium accumulation became evident, with the M5S5 scaffold exhibiting the most intense staining, followed by M0S5, M5S0, and M0S0. Quantitative analysis revealed a statistically significant increase in calcium deposition in the M5S5 group on days 7 and 14 (*p* < 0.05), highlighting its superior biomineralization capacity. The M5S5 scaffold displayed a dense and evenly distributed mineralized matrix, indicating enhanced osteoconductivity. The presence of Sr contributed to sustained osteogenesis, whereas Mg-modulated scaffold dissolution ensured controlled ion release without excessive degradation. Although the M0S5 scaffold also promoted osteogenesis, its effect was slightly weaker than that of M5S5, suggesting that Mg enhances matrix mineralization when combined with Sr [48]. In contrast, M5S0 exhibited relatively weak calcium deposition, confirming that its accelerated degradation limited its long-term mineralization potential. Sustained mineralization on day 14 underscores the role of Sr in enhancing late-stage bone matrix formation, whereas Mg promotes early osteoblast activity. These findings collectively suggest that the co-doping of Sr and Mg in M5S5 provides an optimal balance between bioactivity and degradation, creating a favorable osteogenic microenvironment for bone tissue regeneration. Although the current study focused on in vitro osteogenic responses, these observations are in line with in vivo findings by Nandi et al. [49], who demonstrated that brushite cements co-doped with SrO and MgO enhanced both early (2 months) and late (4 months) phases of bone regeneration. the Mg-doped group exhibited comparable bone mass formation at the early 2-month timepoint compared to the Sr-doped group. However, the Sr-doped group demonstrated a marked increase in bone volume over time, ultimately surpassing the Mg group and indicating its pronounced effect on late-stage bone regeneration and lamellar bone formation. Notably, the Mg/Sr co-doped group exhibited superior new bone formation and osseointegration at both timepoints.

### Pathway analysis of Mg²⁺/Sr²⁺-induced osteogenic and angiogenic responses

**Fig. 10:**
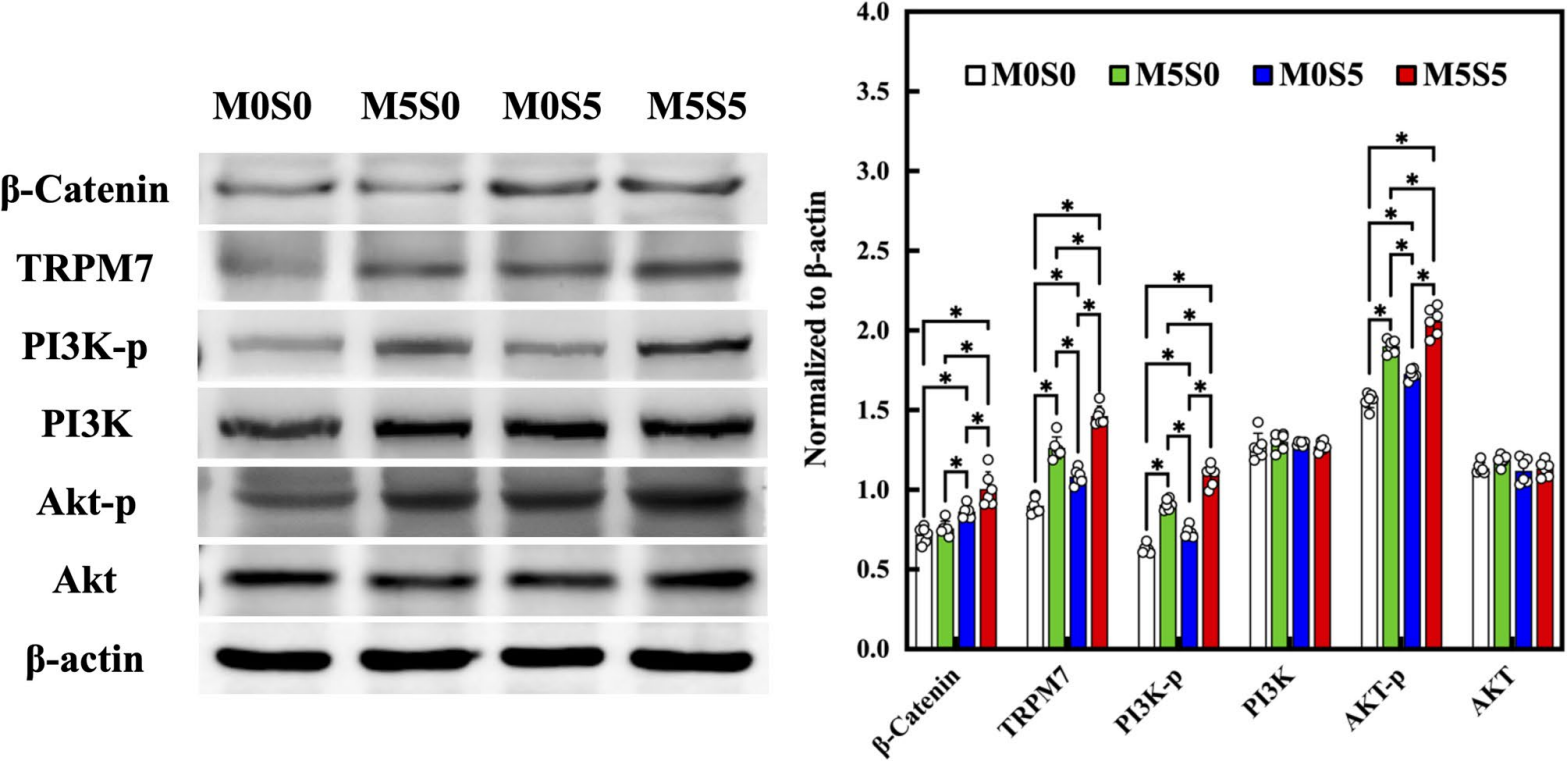
Western blot results of β-catenin, TRPM7, p-PI3K, and p-Akt of WJMSCs cultured on different scaffolds. * indicates a significant difference (*p* < 0.05) between the groups

To further elucidate the molecular mechanisms by which the MSCS scaffolds promote osteogenic and angiogenic differentiation, we investigated the expression of key signaling proteins that have been widely implicated in these biological processes. To this end, β-catenin, PI3K, Akt, and TRPM7 were selected based on their well-established roles in mediating cellular responses to ion-releasing bioceramics [50, 51]. As shown in Fig. 10, the western blot analysis revealed that scaffolds containing Sr^2+^ (M0S5) and Mg^2+^/Sr^2+^ ions (M5S5) exhibited significantly elevated expression of β-catenin compared to the other groups. These findings are consistent with previous studies on Sr-doped bioceramics, which have shown that Sr²⁺ can act as a crucial regulator of MSCs proliferation and osteogenic differentiation by activating the Wnt/β-catenin pathway [52]. Despite existing literature demonstrating that Mg²⁺ can stimulate osteogenic differentiation of MSCs via activation of the Wnt/β-catenin pathway [53], negligible effects were observed in our study. This discrepancy may be attributed to the suboptimal extracellular concentration of Mg²⁺ released from the MSCS scaffolds, which might be insufficient to reach the threshold required for Wnt pathway activation under our specific in vitro conditions. Nevertheless, the Wnt signaling pathway remains a central regulator of embryonic development, organogenesis, and bone remodeling, and is widely implicated in the proliferation and osteogenic differentiation of mesenchymal stem cells. Notably, Sr-mediated activation of Wnt/β-catenin signaling has been associated with enhanced resistance to apoptosis through PI3K/Akt activation, suggesting a mechanistic link between Sr-induced canonical Wnt signaling and the PI3K/Akt pathway in WJMSCs [50].

In contrast, TRPM7, a multifunctional transmembrane protein with both cation channel and kinase activity, plays a key role in intracellular Mg^2+^ homeostasis and regulates the transport of divalent cations, including Mg^2+^, Sr^2+^, and Ca^2+^ [51]. Electrophysiological evidence indicates that PI3K/Akt signaling can modulate cell migration and adhesion by activating TRPM7 in an Mg^2+^-dependent manner. Western blotting results showed that TRPM7, p-PI3K, and p-Akt expression was significantly upregulated in the Mg-containing groups (M5S0 and M5S5) compared to that in M0S5 (*p* < 0.05). It has been well-established that TRPM7 regulates intracellular Mg²⁺ influx and modulates downstream PI3K/Akt and MAPK pathways. For example, Recent evidence further supports the crucial role of TRPM7 in mediating the osteogenic effects of Mg-containing bioceramics. For example, Gong et al. [54] demonstrated that calcium magnesium phosphate cements functionalized with alginate sodium significantly promoted in vitro and in vivo bone regeneration through activation of the TRPM7/PI3K/Akt signaling axis. Their findings highlighted that Mg²⁺ released from the bioceramics upregulated TRPM7 expression and subsequently enhanced the phosphorylation of PI3K and Akt, leading to increased osteogenic marker expression and mineralization [18]. These insights provide mechanistic context to our observation that TRPM7 is significantly upregulated in Mg/Sr co-doped scaffolds (M5S5 group), reinforcing the hypothesis that Mg²⁺ acts through TRPM7-mediated intracellular signaling to potentiate early-stage osteogenic differentiation. Furthermore, when Sr²⁺ is co-present, it may influence the ionic microenvironment in a way that sustains or enhances this TRPM7-driven pathway, thereby amplifying bone regenerative effects. Our previous study demonstrated that a 3D-printed biphasic calcium silicate scaffold composed by Mg-doped CS/PCL and Sr-doped CS/PCL effectively promoted both osteogenic and angiogenic responses by enhancing the expression of Wnt3a, β-catenin, TRPM7, and PI3K/Akt in MSCs, leading to improved bone matrix deposition and vascular network formation. These findings provided key evidence that the dual incorporation of Sr and Mg into calcium silicate scaffolds elicits a synergistic biological response rather than a merely additive effect, supporting the rationale for continued development of MSCS-based biomaterials for bone tissue engineering.

Magnesium ions are well recognized for their pro-angiogenic effects attributed to the ability of stimulating NO production, enhance VEGF secretion, and modulate intracellular calcium flux, all of which are critical for vascular development and homeostasis [12, 15]. Recent studies have revealed that the activation of specific intracellular signaling pathways, plays a pivotal role in promoting angiogenic cytokine production such as VEGF and Ang-1. In parallel, the canonical Wnt/β-catenin pathway, long recognized for its role in osteogenesis, has also emerged as a potential regulator of angiogenesis. Wnt/β-catenin activation in endothelial cells enhances VEGF expression and promotes vascular stability and maturation, partly through cross-talk with PI3K/Akt and MAPK pathways [55]. Notably, Sr²⁺ ions have been implicated in activating Wnt/β-catenin signaling, thus providing a separate but complementary angiogenic stimulus. In our study, scaffolds co-doped with Mg²⁺ and Sr²⁺ (M5S5 group) exhibited a significantly stronger pro-angiogenic response than single-ion groups, as evidenced by enhanced endothelial tube formation and elevated secretion of VEGF and Ang-1. This functional outcome was accompanied by the upregulation of both TRPM7 and β-catenin, suggesting simultaneous activation of Mg- and Sr-related signaling pathways. Collectively, these findings indicate that the co-release of Sr^2+^ and Mg^2+^ exerts a synergistic effect on PI3K/Akt pathway activation through distinct interactions with Wnt and TRPM7 signaling, thereby promoting osteogenic differentiation in WJMSCs and angiogenic capacity of HUVECs. However, to substantiate these mechanistic insights, further studies involving targeted silencing of Wnt3a and TRPM7 using siRNA or specific inhibitors are necessary to clarify their causal roles in mediating the observed effects.

### In vivo analysis of bone regeneration in a rabbit model of critical-sized bone defects

**Fig. 11:**
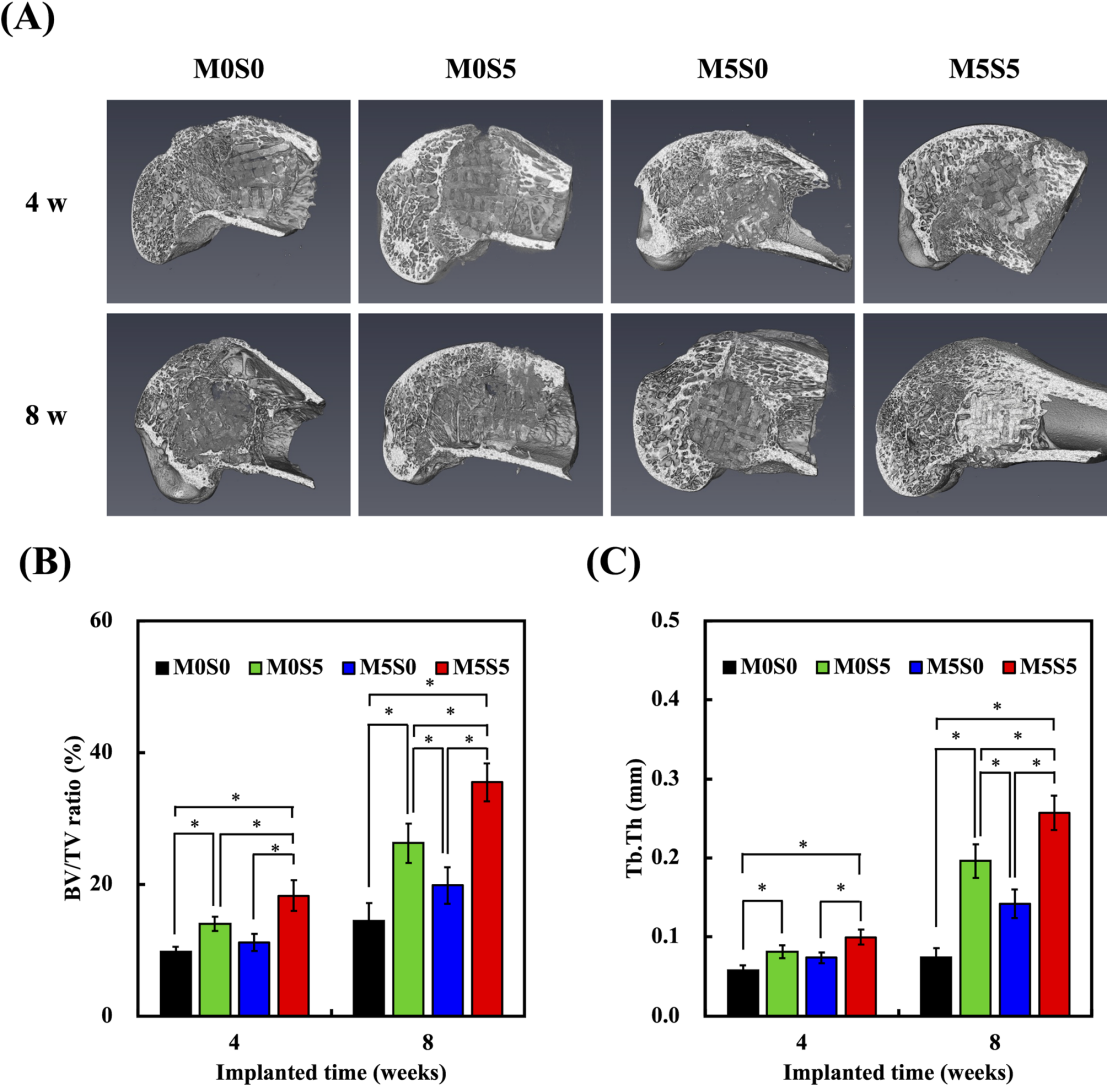
(**A**) Micro-computed tomography images of rabbit femoral defects implanted with MSCS scaffolds at 4 and 8 weeks. (**B**) Bone volume fraction (BV/TV) and (**C**) trabecular thickness (Tb.Th) at 4 and 8 weeks post-implantation. Data are presented as the means ± standard error of the mean; *n* = 6 for each group. The symbol “*” denotes statistically significant differences (*p* < 0.05) between the groups

To evaluate the in vivo osteogenic performance of the scaffolds, µ-CT analysis was conducted at 4- and 8-weeks post-implantation using a rabbit femoral defect model (Fig. 11A). The reconstructed µ-CT images revealed progressive bone formation within the scaffold pores, with the M5S5 group exhibiting the most pronounced new bone growth among all scaffolds investigated. At four weeks, minimal bone formation was observed in the M0S0 group, whereas the M0S5 and M5S0 groups showed moderate levels of bone ingrowth. By week 8, all groups displayed an increased bone volume, with M5S5 demonstrating the most extensive trabecular connectivity, indicating sustained osteogenic activity. Quantitative analysis of the bone volume fraction (BV/TV) (Fig. 11B) showed a time-dependent increase in all the groups, with M5S5 exhibiting the highest BV/TV ratio at 4 and 8 weeks. At 4 weeks, the BV/TV ratio in M5S5 was significantly higher than that in M0S0 (*p* < 0.05), while at 8 weeks, M5S5 maintained significantly greater bone formation than M0S5 and M5S0 (*p* < 0.05). Trabecular thickness (Tb.Th) analysis (Fig. 11C) corroborated these findings, with M5S5 exhibiting significantly greater Tb.Th values than M0S0 at 4 and 8 weeks (*p* < 0.05). Although M0S5 also promoted bone formation, its effectiveness was slightly lower than that of M5S5, reinforcing the beneficial role of Sr in osteogenesis. In contrast, M5S0 displayed the weakest bone regeneration potential, suggesting that its accelerated degradation limited its ability to provide sufficient structural support for new bone ingrowth. The combined µ-CT results highlight the synergistic effects of Sr and Mg in M5S5, which enhanced both early and sustained bone formation. Sr contributed to osteoblast activity and mineral deposition, while Mg regulated scaffold degradation and prevented premature resorption [56]. The controlled degradation of M5S5 ensured the sustained release of bioactive ions, supporting prolonged bone regeneration [57]. These findings suggest that M5S5 provides an optimal balance between bioactivity, degradation, and mechanical stability.

**Fig. 12:**
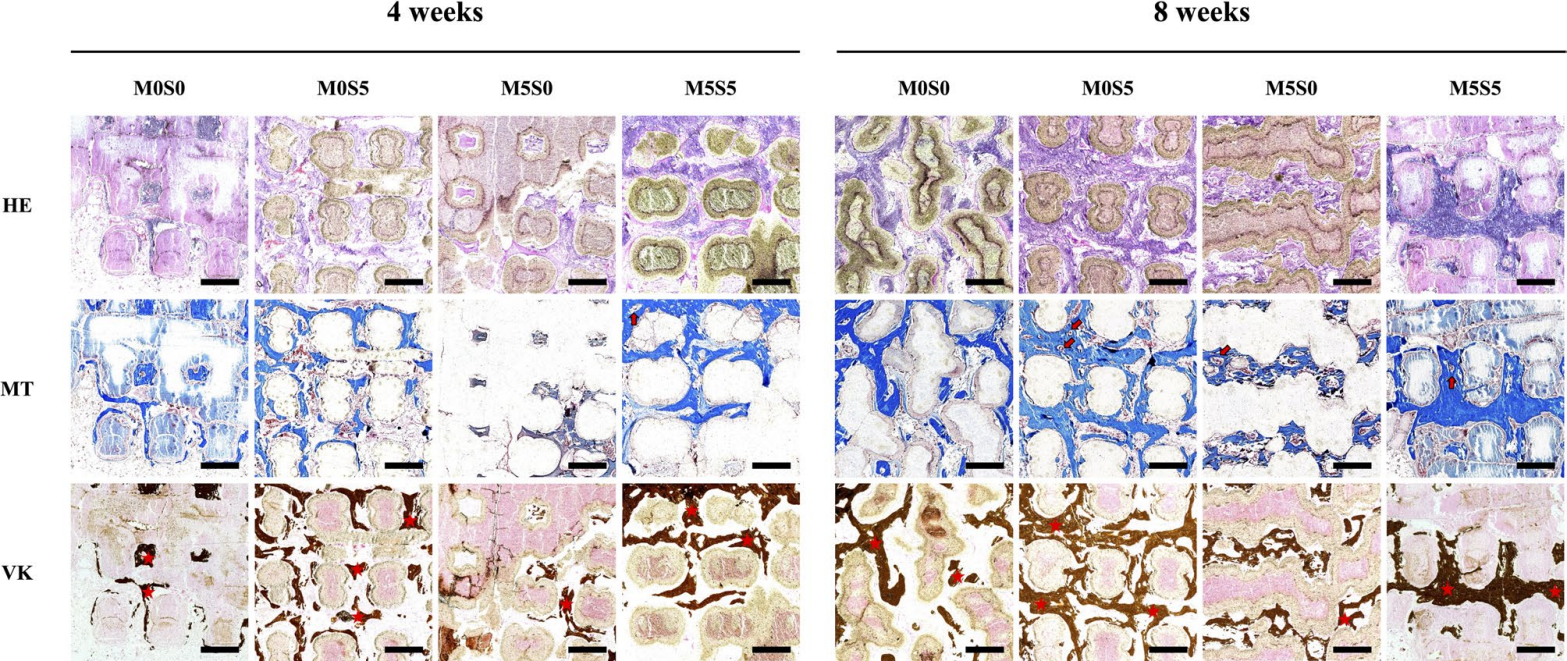
Hematoxylin and eosin, Masson’s trichrome, and Von Kossa staining of rabbit femoral defects implanted with MSCS scaffolds at 4 and 8 weeks post-implantation. Scale bar: 500 μm. Red stars indicate newly formed bone tissue; red arrows highlight newly formed blood vessels

To further evaluate bone formation within the implanted scaffolds, histological staining was performed using HE, MT, and VK staining (Fig. 12). HE staining revealed distinct tissue responses among groups. In the M0S0 group, fibrous tissue dominated the defect site, with minimal new bone formation. In contrast, M0S5 and M5S0 exhibited increased bone matrix deposition, with M5S5 showing the most pronounced bone formation with well-organized trabeculae and limited residual scaffold material. The results of MT staining, which highlights collagen fibers in blue, indicated substantial extracellular matrix formation in the M5S5 group compared to the other groups. The presence of denser collagen structures in the M5S5 scaffolds suggests enhanced osteoid formation, aligning with the µ-CT findings of increased trabecular thickness. In contrast, M0S0 displayed weaker collagen staining, reflecting lower osteogenic activity. VK staining was then used to assess the mineralization levels, with dark staining indicating calcium deposition. The M5S5 group exhibited the most intense mineralization, with extensive dark staining distributed throughout the scaffold, suggesting robust bone formation [58]. M0S5 and M5S0 also showed mineral deposition, although to a lesser extent. M0S0 had the lowest mineralization level, reinforcing its limited osteogenic potential. These histological findings confirmed that the combined presence of Mg and Sr in the CS scaffolds significantly enhanced bone formation, matrix development, and mineralization [59, 60]. The superior performance of M5S5 further supports its potential to promote in vivo bone regeneration. Moreover, the superior in vivo performance of M5S5 suggests that the combined presence of Mg and Sr ions plays a crucial role in facilitating osteogenesis and promoting mineralization while maintaining sufficient mechanical stability for prolonged bone regeneration.

## Conclusion

This study confirmed the osteogenic and angiogenic potential of 3D-printed Mg/Sr co-doped CS scaffolds and highlighted their capacity to serve as advanced biomaterials for bone tissue engineering. Incorporating Sr and Mg into a calcium silicate matrix effectively modulates the phase composition, ionic dissolution kinetics, and degradation profiles, leading to a finely tuned balance between scaffold bioactivity and structural integrity. Specifically, the M5S5 scaffold exhibited enhanced osteoinductivity and angiogenic capacity, as evidenced by increased alkaline phosphatase activity, extracellular matrix mineralization, and endothelial network formation. In vivo µ-CT and histological analyses further confirmed the superior bone regeneration performance of the M5S5 group, which was characterized by increased bone volume fraction, trabecular thickness, and collagen deposition. These findings underscore the critical role of synergistic bioactive ion release in orchestrating cellular responses and provide a rational design strategy for next-generation bone scaffolds that integrate osteogenesis and vascularization to optimize skeletal repair and regeneration.

## Data Availability

No datasets were generated or analysed during the current study.
